# Assessing changes in range of motion in adolescent patients undergoing myoActivation® for chronic pain related to myofascial dysfunction: a feasibility study

**DOI:** 10.3389/fpain.2023.1225088

**Published:** 2023-10-25

**Authors:** Tim Bhatnagar, Farah T. Azim, Mona Behrouzian, Karen Davies, Diane Wickenheiser, Gail Jahren, Nicholas West, Lise Leveille, Gillian R. Lauder

**Affiliations:** ^1^ The Motion Lab, Sunny Hill Centre at BC Children’s Hospital, Vancouver, BC, Canada; ^2^ Department of Pediatrics, Faculty of Medicine, University of British Columbia, Vancouver, BC, Canada; ^3^ School of Biomedical Engineering, University of British Columbia, Vancouver, BC, Canada; ^4^ Department of Physical Therapy, Faculty of Medicine, University of British Columbia, Vancouver, BC, Canada; ^5^ Department of Nursing, BC Children’s Hospital, Vancouver, BC, Canada; ^6^ Research Institute, BC Children’s Hospital, Vancouver, BC, Canada; ^7^ Department of Orthopedics, University of British Columbia, Vancouver, BC, Canada; ^8^ Department of Anesthesiology, Pharmacology and Therapeutics, University of British Columbia, Vancouver, BC, Canada

**Keywords:** myoActivation, chronic pain, myofascial dysfunction, motion capture, adolescent

## Abstract

**Introduction:**

myoActivation® assessment utilizes systemized movement tests to assess for pain and limitations in motion secondary to myofascial dysfunction. myoActivation needling therapy resolves the myofascial components of pain and is associated with immediately observed changes in pain, flexibility, and range of motion. The principal aim of this feasibility study was to objectively characterize the kinematic metrics of upper and lower body motion before and after myoActivation movement tests and therapy.

**Methods:**

Five consecutive eligible adolescent participants considered appropriate for myoActivation were consented to receive their myoActivation intervention in a motion laboratory. Clinical motion analysis was used to measure the changes in maximum range of motion (maxROM) and maximum angular speed to maximum ROM (speedROM) of movement tests predicted to change. Metrics were analyzed to assess changes over specified time intervals - i) baseline to after initial myoActivation session, and ii) baseline to after complete myoActivation course. Each participant served as their own control.

**Results:**

We demonstrated objective evidence of improved maxROM and/or speedROM in 63% of the movement tests predicted to change after just one session of myoActivation and in 77% of movement tests predicted to change over the complete course of treatment. The myoActivation clinician observed positive change in 11/19 of movement tests across all patients, that were predicted to change after the initial myoActivation session; 81% of these positive changes were confirmed by the kinematic data.

**Discussion:**

Clinical motion analysis provides objective support to clinicians evaluating, treating, and teaching myofascial release. A larger, prospective clinical trial is warranted to explore the impact of myoActivation on movement. Refinement of observation techniques and outcome measures established in this feasibility study will strengthen future clinical motion analysis of the myoActivation process.

## 1. Introduction

Chronic myofascial pain (CMP) is common in the pediatric population (1–3). It combines myofascial dysfunction and musculoskeletal pain (4, 5) and is characterized by deep pain in a non-dermatomal distribution, peripheral sensitization, referred pain, and central sensitization (6). The associated myofascial stiffness also generates anomalous tension, affecting the whole fascial continuum, which leads to progressive immobility (7, 8).

Myofascial dysfunction can be identified by detecting a combination of one or more of the following physical findings: trigger points in muscles, fascial changes, scars, changes in posture, and/or altered perceived weight distribution between the feet. Muscular trigger points (MTrPs) are irritable nodules, located predominantly near the motor end plates in taut bands of skeletal muscle (9). Fascial trigger points (FTrPs) are palpable densities (palpable pain points in fascia) often located near active MTrPs (10, 11). Scars have implications that reach beyond their aesthetic appearance. Scars can cause local and distant effects related to restricted movement of underlying tissues (12). The presence of these MTrPs, FTrPs, and scars are associated with stiffness and restricted range of motion, which generates anomalous tension that affects the whole fascial continuum (13, 14).

As all structures of the human body are intricately connected through the skin and the myofascial system, myofascial release of MTrPs, FTrPs, and scars can result in improved pain, flexibility, and range of motion (ROM) (12). Myofascial release techniques are used in manual medicine to restore the optimal length of myofascial tissues to improve function, release tension and improve pain (15). Myofascial release can affect ROM, not just locally, but at distant sites too, through the impact of myofascial chains (12); for example, treatment of the plantar fascia results in improved hip ROM as well as increased hamstring flexibility, and stretching of the hamstring muscles leads to increased cervical spine flexibility through the connections in the posterior fascial chain (16).

**Figure 1:**
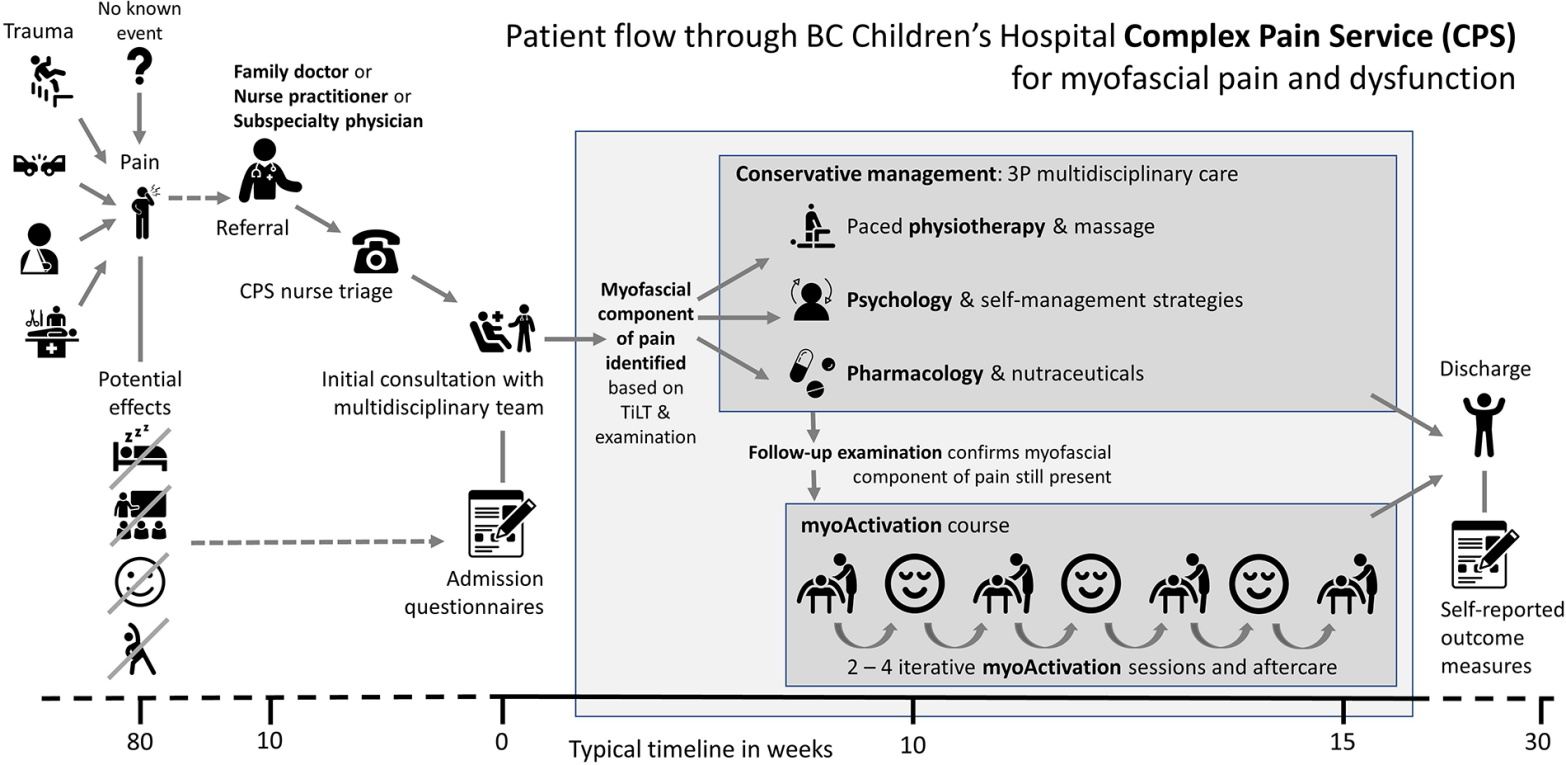
Flow through BC Children's hospital complex pain service for patients with myofascial pain and dysfunction.

**Figure 2:**
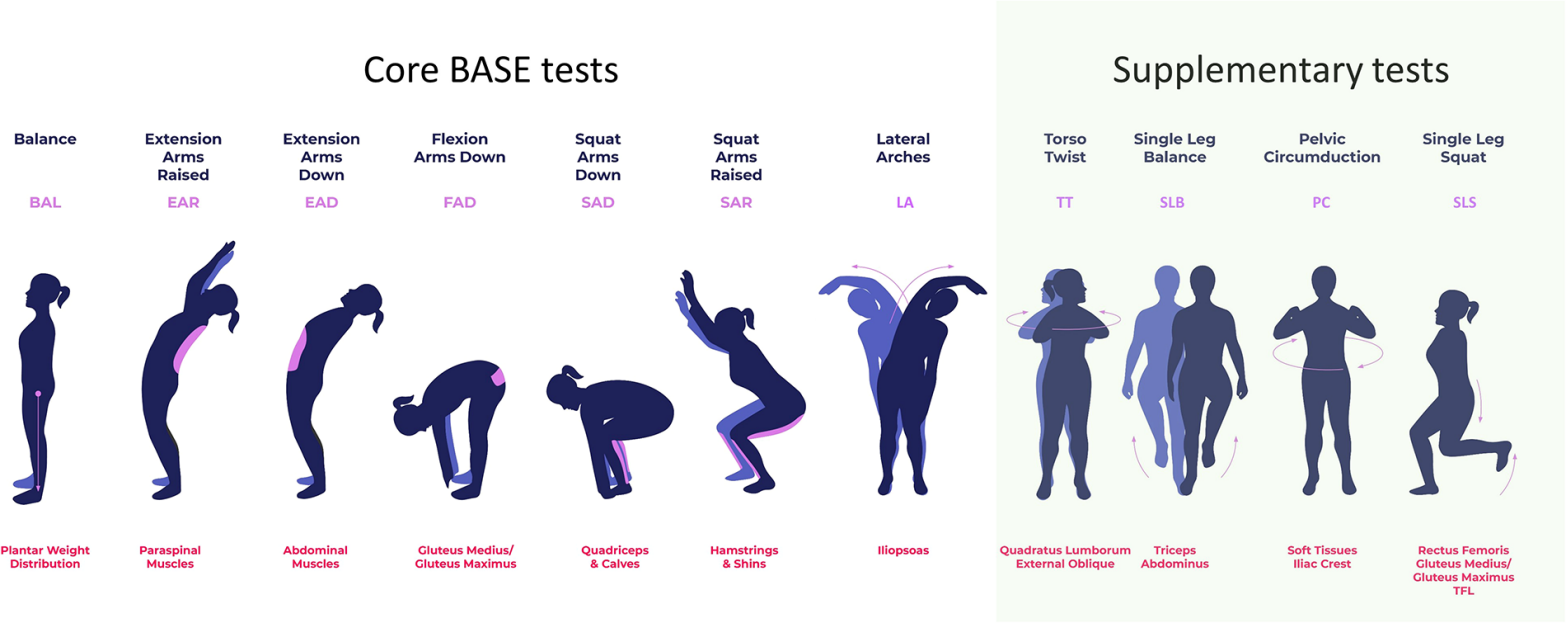
The seven core BASE myoActivation movement tests and additional basic movement tests that are used in myoActivation assessment. Each movement test is labelled at the top, with its abbreviation in purple; the purpose of each test, including muscle groups evaluated, is indicated in red text at the bottom.

When a myofascial component of pain or dysfunction is diagnosed, myoActivation® (6, 17) can be utilized as part of a multidisciplinary approach to chronic pain management (Figure 1). myoActivation is a novel structured process of assessment and treatment that recognizes and treats myofascial components of chronic pain (17). Preliminary evidence for myoActivation has been published in case series describing its use and effectiveness in both adults (17, 18) and children (6); it is the subject of ongoing research (19) and structured educational program (20). The myoActivation process includes a comprehensive timeline of lifetime trauma (TiLT) history; a unique, quick, structured physical assessment, based on a systematized and repeatable set of movement tests (Figure 2). myoActivation therapy entails refined trigger point injections to restore anatomic integrity to injured tissues. Three myofascial interventions are integrated in the myoActivation process; muscle trigger point injections, fascial release, and scar release. Very fine gauge hypodermic needles are inserted into trigger points that compromise function of muscle, fascia, and/or scar tissue. Many studies support the use of trigger point injections for myofascial dysfunction and pain (21–24). Needling without an injectate is as equally effective as injection of anesthetic agents (25–27). myoActivation results in immediately observed changes in pain, flexibility, ROM, and more balanced perceived weight distribution between the feet (6). These changes are all noted at the time of treatment through observed changes and cyclical repetition of the standard movement tests.

Clinical motion analysis (CMA) is the systematic study of human motion using observed and measured body movements, body mechanics, and the activity of muscles. In this study, instrumented motion analysis—performed in The Motion Lab, Sunny Hill Health Centre at BC Children's Hospital (TML)—was utilized to capture joint kinematics during myoActivation assessments. Joint kinematics can be used to characterize aspects of movement including the magnitude of motion and speed of motion. CMA has been utilized in other studies when assessing how chronic pain can affect altered kinematics and postures (28, 29); however, it has not previously been used to quantify the kinematic effects of myoActivation.

This feasibility study was designed to objectively measure the changes in motion that myoActivation physicians observe following myoActivation therapy. Hence, we aimed to: (1) evaluate the feasibility of conducting myoActivation assessment and treatment sessions in TML; (2) determine the kinematic variables that reflect changes in ROM, and speed of motion over specified time intervals, including—(i) baseline to after initial myoActivation session, and (ii) baseline to after complete myoActivation course—in adolescent patients undergoing myoActivation treatment for CMP; (3) use this quantitative data to inform and power a clinical trial to demonstrate changes in movement kinematics in adolescent patients undergoing myoActivation as part of their care for CMP.

## 2. Materials and methods

### 2.1. Study design

This was a non-randomized feasibility study of five patients undergoing myoActivation assessment with CMA. The study group received standard myoActivation therapy, with some of these sessions performed in TML. Participants were assessed with CMA while executing myoActivation assessments—seven Biomechanical Assessment and Symmetry Evaluation (BASE) tests and four others (Figure 2). CMA was conducted prior to the initial myoActivation session (“baseline”) and then after each intervention that was performed in TML. The final CMA was conducted at the very end of the patient's myoActivation course of treatment. There was no control group as each study patient served as their own control.

The study was approved by the University of British Columbia/Children's and Women's Health Centre of British Columbia Research Ethics Board (H20-00463, principal investigator G R Lauder, approval date 09 Mar 2022). This manuscript has been prepared in accordance with the STROBE (STrengthening the Reporting of OBservational studies in Epidemiology) guidelines (30).

### 2.2. Setting

British Columbia's Children's Hospital (BCCH) is a tertiary care center that provides care for children and adolescents across British Columbia. The BCCH Complex Pain Service (CPS) is a multidisciplinary service that takes a holistic approach to pain management and individualizes treatment to each patient's specific needs and goals. Children and adolescents referred to the CPS have often seen many physicians and undergone many investigations to exclude a remedial cause for their pain. After full review of the pain history, timeline of lifetime trauma (TiLT) and myoActivation examination, many of these children and adolescents are diagnosed with a myofascial component to their pain (Figure 1). The CPS has utilized myoActivation since July 2017 as part of their multidisciplinary approach (6).

The Motion Lab, Sunny Hill Health Centre at BCCH (TML) is a state-of-the-art facility for conducting CMA. TML performs CMA using a Qualisys 12-camera passive reflective marker motion capture system (Qualisys, Göteborg, Sweden) in combination with synchronized floor-embedded force plates (1,200 Hz; AMTI, Allentown, MA). This system records the 3D location of reflective markers affixed to the body at specified anatomical landmarks and uses them to generate a full body model for kinematic and kinetic analysis.

TML is able to facilitate clinical procedures (e.g., myoActivation therapeutic interventions) within the lab environment in patient private preparation rooms located next to the motion capture area. CMA data from clinical and research activities are stored in TML database on password-protected computers, according to methodologies outlined in Research Ethics Board approved protocols.

### 2.3. Terminology

The following terminology will be used to describe the myoActivation approach and study activities: *assessment* indicates a full set of movement tests (Figure 2); *intervention* indicates the needling technique used by the CPS physician as directed by myoActivation principles (17) and usually consists of needling at multiple sites; *session* indicates all the assessments and interventions conducted on a single day for each participant (there will be one more assessment than intervention in each session, with at least two assessments and one intervention in each session, but typically more than this); finally, *course* indicates all the sessions completed for any one participant throughout the study period.

The following abbreviations have been used in this manuscript: BASE (Biomechanical Assessment and Symmetry Evaluation), BCCH (British Columbia Children's Hospital), CMA (clinical motion analysis), CMP (chronic myofascial pain), CPS (Complex Pain Service), FTrP (Fascial trigger point), HE (hip extension), HF (hip flexion), maxROM (maximum range of motion, defined as the maximum angle from the neutral angle), MTrP (Muscular trigger point), ROM (range of motion), speedROM (maximum angular speed towards maximum range of motion) TiLT (timeline of lifetime trauma), TML (The Motion Lab), TWRP-E (thorax with respect to pelvis—extension), TWRP-F (thorax with respect to pelvis—flexion). Also, the following abbreviations are used for each of the myoActivation movement tests (Figure 2): BAL (balance), EAR (extension arms raised), EAD (extension arms down), FAD (flexion arms down), SAD (squat arms down), SAR (squat arms raised), LA (lateral arches), PC (pelvic circumduction), SLB (single leg balance), SLS (single leg squat), TT (torso twist).

### 2.4. Participants

Eligible participants included adolescent patients (≥14–≤19 years), American Society of Anesthesiologists' Physical Status I–III, who had been diagnosed with chronic myofascial dysfunction. Exclusion criteria were: abnormal developmental profile; and fear or aversion of a needling technique. Following assessment by the CPS multidisciplinary team, consecutive eligible patients, who had been diagnosed with myofascial dysfunction, and had undergone a period of conservative management, were approached and informed about this study.

These participants were considered appropriate to be able to participate in myoActivation. Participants were not recruited or consented for myoActivation if they had chronic pain which was not related to myofascial dysfunction or they met either of the exclusion criteria. All participants had previously provided informed consent to receive myoActivation. Consent for this study was to perform objective measurement of the structured myoActivation movement tests in TML.

CPS patients with myofascial dysfunction are typically expected to need 2–4 myoActivation sessions, usually scheduled one week apart; this may vary depending on the family's location and availability. Each session typically lasts 30 min to one hour, including preparation and consultation time. Continued study participation was verbally confirmed with the participant and any accompanying family member at the beginning of each myoActivation session.

### 2.5. Outcomes

In the current study, two intervals were used to analyze changes in motion during execution of the movement tests that were expected to change due to intervention: (i) baseline to after initial myoActivation session, and (ii) baseline to after complete myoActivation course. The clinician predicted the movements expected to change based on the myofascial areas that were treated; these expectations were recorded at the time of the treatment and the clinician did not have access to the kinematic data at this stage.

The primary outcome was (a) quantified changes in maximum ROM (maxROM) of involved joints in the pertinent anatomical plane (i.e., sagittal, coronal or transverse plane) kinematics. Secondary outcomes were: (b) quantified change in maximum angular speed to maximum ROM (speedROM) of involved joints, from collected kinematic data and (c) changes in self-reported pain. Changes in measured maxROM and speedROM were considered clinically significant if the change due to intervention resulted in an increase of 5° or 5°/s, respectively.

### 2.6. Study procedures

A trained physiotherapist placed 43 passive-reflective markers on pre-specified, anatomically relevant landmarks on each participant, according to the Qualysis marker system.

After system-patient calibration, baseline CMA measurements were taken of the participant performing a structured set of 11 movement tests: seven core BASE tests (balance [BAL], extension arms raised [EAR], extension arms down [EAD], flexion arms down [FAD], squat arms down [SAD], squat arms raised [SAR], and lateral arches left/right [LA-L, LA-R]) and four supplementary tests (torso twist left/right (TT-L, TT-R], single leg balance left/right [SLB-L, SLB-R], pelvic circumduction [PC], and single leg squat left/right [SLS-L, SLS-R]) (Figure 2). For clarity, the results for movement tests that are repeated for left and right movements (-L and -R), for example LA-L and LA-R, are given separately, but counted as a single movement test in our analysis.

The CPS physician followed a script (see Appendix A) to instruct participants on how to perform myoActivation movements and to inquire if participants felt any pain/discomfort during the movement, along with the site of that pain that the movement provoked; the pain report was simply a response to the question “What are you feeling?” or “Any tension or discomfort?” depending on the movement. The CPS team recorded observations of each participant's performance and responses. Motion capture was performed during each set of movement tests that occurred in TML.

Following completion of initial movement tests in TML (this being the third time participants would have done these movement tests since their initial contact with the CPS team), the participant underwent myoActivation intervention, based on the CPS team's interpretation of their observations of the movement tests and the participant feedback. Interventions were performed in a private examination room separate from the TML cameras and staff. The CPS team recorded the movements predicted to change based on the myofascial tissues that were treated. After intervention, the participant then performed all movement tests again, as previously described, while kinematic data were acquired.

Participants received a course of myoActivation treatments over a different number of individually assessed sessions. Participants were provided with myoActivation intervention, even if TML was not available to collect their motion capture data, to ensure they did not miss planned treatment sessions, as determined by the CPS team. Therefore, CMA was not utilized for every session, but the key timepoints in the current study of “before the initial intervention” (i.e., baseline), “after the initial myoActivation session” and “after the course of myoActivation sessions” were completed in TML for all participants. The CPS physician determined the number and location(s) of the myoActivation sessions provided to each participant.

### 2.7. Data analysis

**Figure 3:**
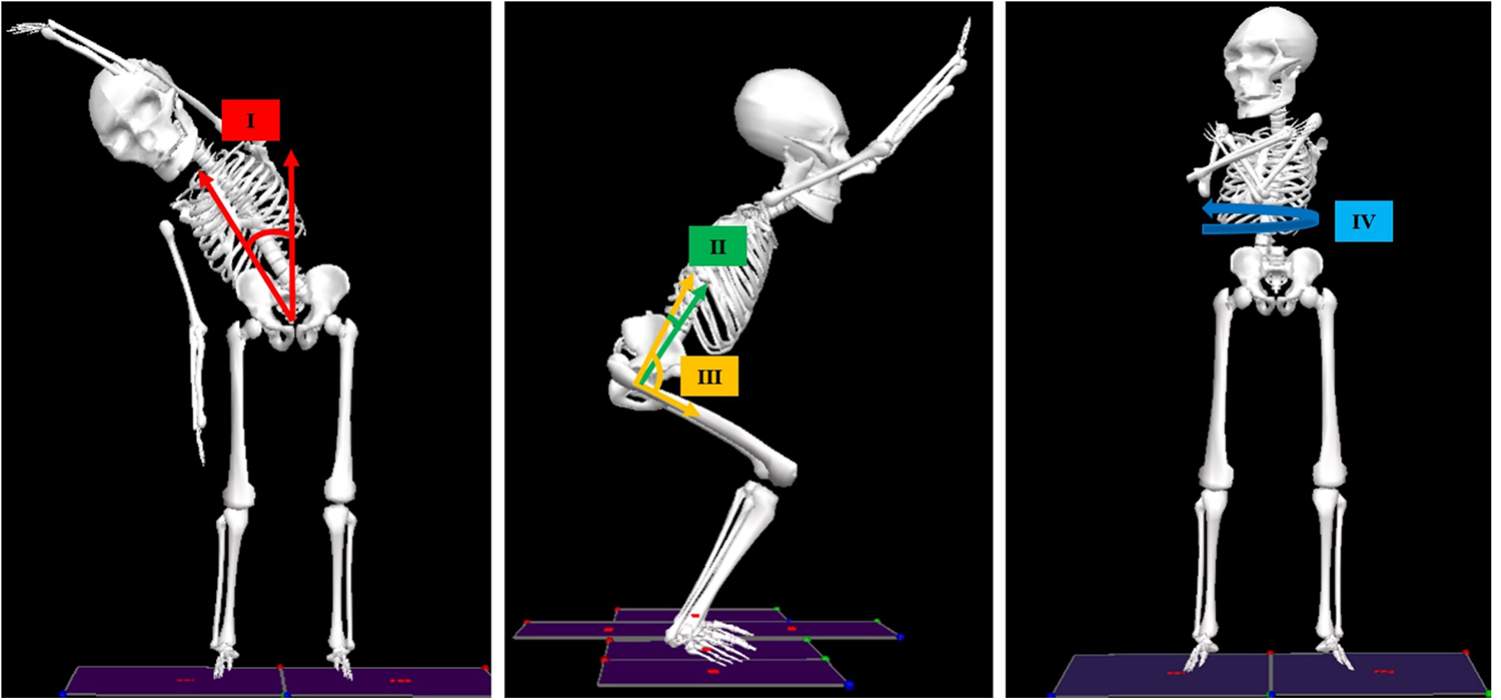
Joint angles analyzed in the current study. Left: Thorax-with-respect-to-pelvis (TWRP) bend angle in the coronal plane (**I**); Middle: TWRP flexion/extension (**II**) and hip flexion/extension (**III**) in the sagittal plane; Right: TWRP rotation in the transverse plane (**IV**).

Each movement test was captured using the TML hardware previously described, as well as with Qualisys Track Manager (QTM) with the Project Automation Framework Functional Assessment package (Qualisys AB, Göteborg, Sweden) and analyzed using Visual 3D (C-Motion, Germantown, MD). To quantify the movement of the participants, coordinate systems were established for the following body segments: thorax, pelvis, and for each leg—thigh, shank, and foot. Calculation of three-dimensional angles between segments (i.e., “joint angles”) was part of the analysis performed in Visual3D (31). The participant motion presented in the current study is determined by comparing orientations of adjacent segments [e.g., the “thorax with respect to pelvis” (TWRP) angle]. For clarity, this manuscript will discuss a subset of the eleven movement tests: EAR, EAD, FAD, SAD, SAR, LA (-L and -R), and TT (-L and -R) (Figure 2). Participant body motion was analyzed for each movement test in either the sagittal, coronal or transverse planes, depending on which plane captured the prescribed movement (Figure 3). Relevant joint angles were then plotted against time. Three data sets are plotted on each graph: (i) baseline, (ii) after initial myoActivation session, and (iii) after complete myoActivation course. For analyses of movement of bilateral joints (e.g., hip flexion), the average of the left and right joint kinematic data was used.

**Figure 4:**
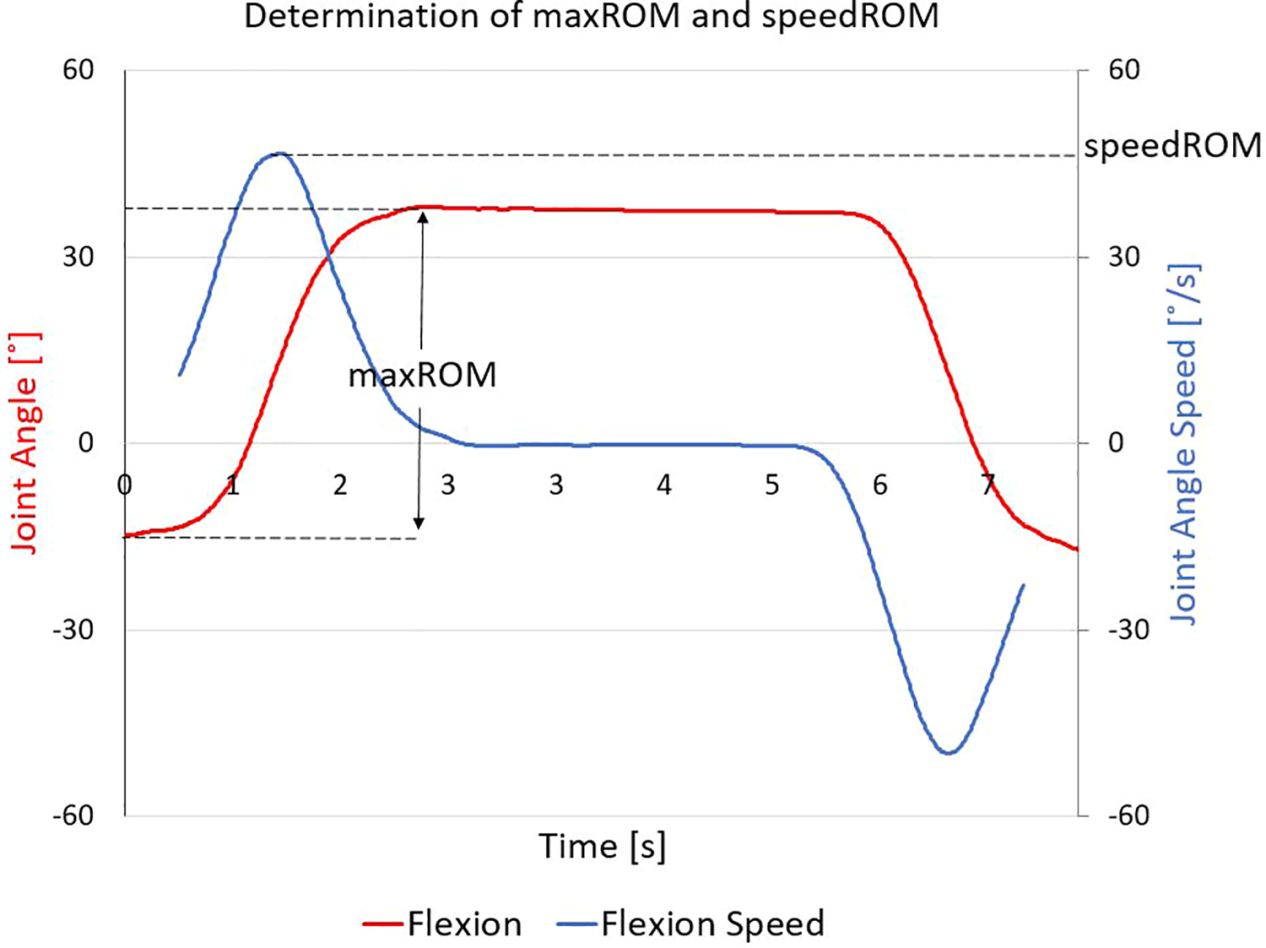
Determining maximum range of motion (*maxROM*) and estimated maximum angular speed towards maxROM (*speedROM*): this representative dataset shows flexion (red line), from which the *maxROM* is determined as the magnitude of difference between neutral posture and maximum flexion; the filtered first time-derivative (blue line) is calculated, from which *speedROM* is determined as the absolute value of the local maxima during the interval wherein the participant is moving towards their *maxROM*.

For each participant, two characteristics were derived from the kinematic data for each movement test and compared over the two intervals of interest: (1) the quantified change in maximum range of motion (maxROM—defined as the maximum angle from the neutral angle), and (2) the quantified change in estimated maximum angular speed towards maximum ROM (speedROM). speedROM was determined by taking the first-derivative of the planar joint angles over time, utilizing an averaging filter to reduce signal noise (window size: 101 data frames), and determining the local magnitude maxima that corresponded to the time interval when the participant was moving towards maxROM (Figure 4). Evident increased maxROM (>5°), and/or evident increased speedROM (>5°/s), for any joints involved in the motion, were interpreted as improvements in movement. These thresholds were set based on reflective marker placement uncertainty metrics, previously established in TML as part of our internal quality assurance process. All CMA labs conduct repeatability testing to determine the ability for their hardware to report kinematic metrics reliably when variation of reflective marker placement is considered; these thresholds can be used, effectively, to indicate evident increases in maxROM and speedROM that can be assumed to not be due to marker placement error.

The kinematic metric changes between baseline and after the initial myoActivation session were compared to evaluate the effects from a single myoActivation session. The kinematic metrics at baseline and the final myoActivation session were compared to evaluate the cumulative changes over the entire course of myoActivation therapy. Participants received different numbers of myoActivation interventions between the initial and final datasets being compared. Participants received individually assessed, but different, myoActivation interventions based on their assessment at each session. Required interventions were determined by the clinician, solely based on clinical observation of the patient performing the prescribed set of movement tests; targeted interventions were not based on the processed kinematic data during this feasibility study as these were not available until after treatment had been administered.

In addition, for the first interval—baseline to after initial myoActivation session—kinematics-based objective evidence of change in movement (i.e., maxROM and/or speedROM) were compared against the CPS physician's qualitative assessment of whether a change in performance of movement tests was observed. Kinematic data were considered to confirm physician observations of improvement if either maxROM or speedROM for any joints assessed for a movement demonstrated improvement. The kinematic data were also compared against the clinician's predictions of which movement tests were expected to change; the clinician had made and recorded these predictions at the time of treatment and before viewing the processed kinematic data. The movement tests for which final TML kinematic metrics were predicted to change were based on the accumulated interventions of the whole myoActivation course.

Due to the small sample size of this study, no statistical comparisons were performed for any of the data collected.

## 3. Results

### 3.1. Participants

Study participants were five adolescent females of median (range) age 16 (16–18) years, weight 63.4 (48.4–105.9) kg, and height 167 (161–175) cm (Table 1). All had experienced chronic pain for more than 1 year prior to their admission to the CPS.

**Table 1:** Participant demographicsand initial presentation.

ID	Demographics			Presentation				Recreational activities	Greatest reported physical trauma	Comorbidities
	Age (years)	Sex	Weight (kg) Height (cm)	Pain site	Pain duration	Timeline of lifetime trauma (TiLT)	Most difficult movement			
1	18	F	63.4 161	Right Hip	4 years	No MVA No fracture No surgery No fall on coccyx	“Moving legs”	Dragon Boat Racer	Hitting right shin on cement bench 6 years before presentation	Anxiety (GAD/seasonal affective disorder) ADHD Mood disorder Panic attacks
2	16	F	48.4 167	Left Lower Quadrant Abdomen	Over 5 years	No MVA No surgery No fracture Fall on coccyx	Running	Soccer Field Hockey	Soccer collision and injury to right knee 4 years before presentation	None
3	16	F	62.6 166	Multisite & CLBP	2 years	No MVA No surgery No fractures No Fall on coccyx	Running Fast walking Lifting backpack	Agility classes Soccer	None reported	None
4	17	F	105.9 175	Left Hip & CLBP	11 years	MVA x 2 with no injuries. Hip arthroscopy No Fall on coccyx	Climbing stairs Sitting	None	None reported	Increased BMI Pes planus Genu valgum
5	16	F	60.5 163	CLBP	1 year	No MVA Surgery for rhadomyosarcomma Bilateral ports No Fall on coccyx	Walking	None	None reported	Bladder Rhadomyosarcomma Rx at 18 months of age. Neobladder Mitrofonoff

CLBP, chronic lower back pain; CPS, complex pain service; TiLT, timeline of lifetime trauma; MVA, motor vehicle accident; GAD, generalized anxiety disorder; ADHD, attention deficit hyperactivity disorder; Rx, treatment.

### 3.2. Myoactivation assessment and treatment

All participants had an initial examination at the time of presentation to the CPS, which included myoActivation movement tests. All participants underwent a period of conservative management to help resolve myofascial dysfunction [median 12 weeks (range 7–20 weeks)]. Repeat myoActivation assessment was performed for the second time after this period of conservative management. If this second assessment indicated that a myofascial component of pain still existed (Table 2), consent was obtained for myoActivation intervention. Participants attended a median (range) of 3 (2–5) myoActivation sessions (Table 3). All had improved outcomes after this program of treatment and 4/5 have now been discharged from the care of the CPS (Table 4).

**Table 2:** Examination findings which confirmed a myofascial component to pain.

ID	Reported weight on feet balanced or NOT	Initial CPS presentation		Initial TML assessment		Scars identified	Fascia in tension identified	Muscles in sustained contraction identified
		BASE test with most restricted ROM	Most painful BASE test	BASE test with most restricted ROM	Most painful BASE test			
1	Not	EAR	SLS-R	SLS-R	SLS-R	Yes	Yes	Yes
2	Not	FAD	EAR	FAD	LA-L/LA-R	Yes	Yes	Yes
3	Not	FAD	EAR	FAD	EAR	No	Yes	Yes
4	Not	EAD	None	EAR	TT-L/TT-R	Yes	Yes	Yes
5	Not	EAR	EAR	EAR	EAR	Yes	Yes	Yes

CPS, complex pain service; ROM, range of motion; EAR, extension arms raised; flexion arms down; FAD, SLS, Single leg squat; LA, lateral arch; TT, torso twist (see Figure 2). Physician observed findings.

**Table 3:** myoActivation treatment details: muscles activated, scars and fascia released for all myoActivation sessions.

ID	Session (weeks after initial session) + location (TML)	Muscles activated	Scars released	Areas where Fascia in tension released
1	Initial, TML	Bilateral iliopsoas, right external oblique	Right shin Left Knee	Right Iliac crest
	+1 week	Bilateral iliopsoas, Bilateral rectus abdominus, right pectineus, right gluteus minimus, right tensor fascia lata		Bilateral pubic fascia, right iliac crest, right ilio-tibial band
	+4 weeks, TML	None	Right knee scar, right shin scar	None
2	Initial, TML	Bilateral iliopsoas, left external oblique, left rectus abdominus	Left Knee scar, Chin scar	Left iliac crest, line of tension above umbilicus
	+2 weeks	Bilateral iliopsoas, bilateral gluteus medius, left rectus abdominus, left external oblique		Left iliac crest
	+12 weeks, TML	Measurement only—no intervention		
3	Initial, TML	Left iliopsoas, right paraspinal, left rectus abdominus, left external oblique, bilateral gluteus medius	None	Right iliac crest, line of tension above umbilicus
	+1 week	Left quadratus lumborum left iliopsoas, right gluteus medius and minimus, right paraspinal, bilateral rectus abdominus, bilateral trapezius, left cervical paraspinal, left scalene		
	+13 weeks, TML	Measurement only—no intervention		
4	Initial, TML	Left quadratus lumborum, bilateral rectus abdominus, left iliopsoas	Left thigh scar	Left flank skin crease, left iliac crest
	+1 week	Left external oblique, bilateral rectus abdominus, right adductor, bilateral vastus medialis, left vastus lateralis.		
	+2 weeks	Right paraspinal, left rectus femoris, left vastus lateralis, left vastus medialis, right gluteus medius	Left thigh scar	
	+5 weeks	Left iliopsoas, right paraspinal, left gluteus minimus	Left knee scar	Left flank skin crease, left iliac crest
	+9 weeks, TML	Right paraspinal, right vastus medialis, right forearm extensors, right brachioradialis		Coccyx, bilateral iliac crest
5	Initial, TML	Right paraspinal	None	Left iliac crest, coccyx
	+1 week		Abdominal scars×4	Coccyx, left pubic fascia
	+1 week	Bilateral paraspinals	Abdominal scars×4	
	+3 weeks, TML	Right paraspinal, left iliopsoas, left vastus medialis	Bilateral port scars Left Neck scar	Linea alba above umbilicus

TML, the motion lab.

**Table 4:** Outcome measures from Complex Pain Service (CPS) care.

Case	Pre-care condition at time of initial assessment					Discharge		Post-care outcomes				
	Pain Yes/No	Physical function impacted Yes/No	Sleep impacted Yes/No	School attendance impacted Yes/No	Mood impacted Yes/No	Discharged from CPS Yes/No	Duration of CPS care	Pain	Physical function	Sleep	School attendance	Mood
1	Yes	Yes	Yes	Yes	Yes	Yes	41 weeks	Improved	Improved	Improved	At college	“Pretty Good”
2	Yes	Yes	Yes	No	Yes	No	ongoing	Improved	Improved	Improved	Same	Improved
3	Yes	Yes	No	No	Yes	Yes	43 weeks	Improved	Improved	Good	At university	Good
4	Yes	Yes	Yes	Yes	No	Yes	30 weeks	Improved	Improved	“Well”	Working	“Pretty good”
5	Yes	Yes	Yes	Yes	Yes	Yes	44 weeks	Improved	Improved	Good	Working	Good

### 3.3. Kinematic data

**Figure 5:**
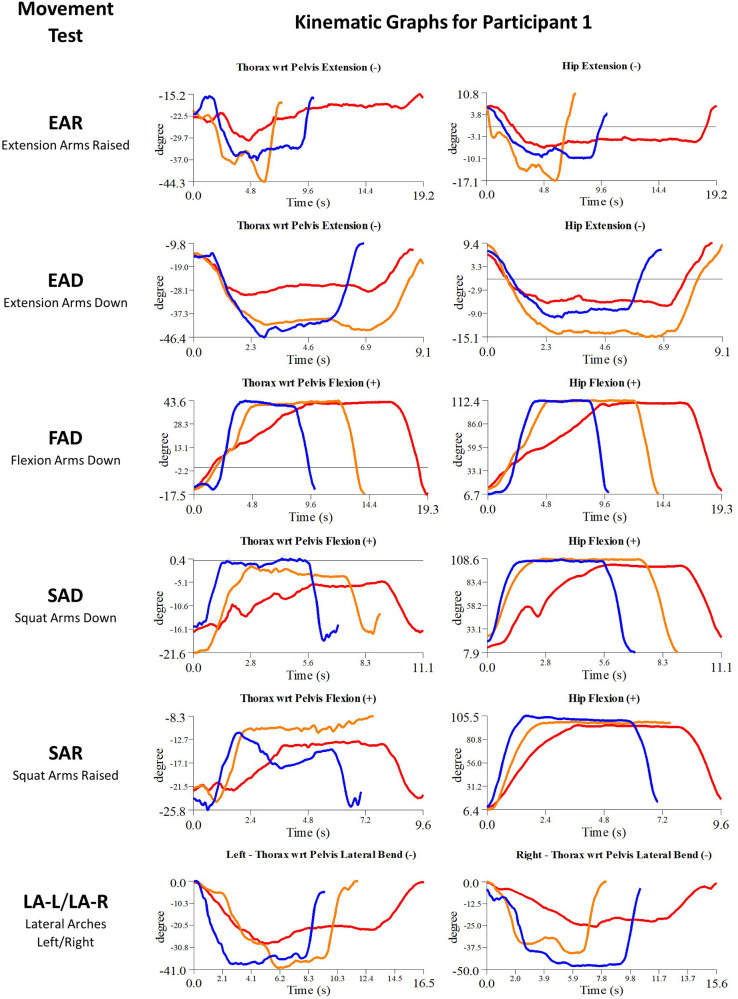
Participant 1 kinematics—single-plane joint angles plotted against time for the movement tests that were expected to change, or demonstrated unexpected changes; (i) baseline(red), (ii) post-first myoActivation session (orange), and (iii) post-final myoActivation session (blue).

**Figure 6:**
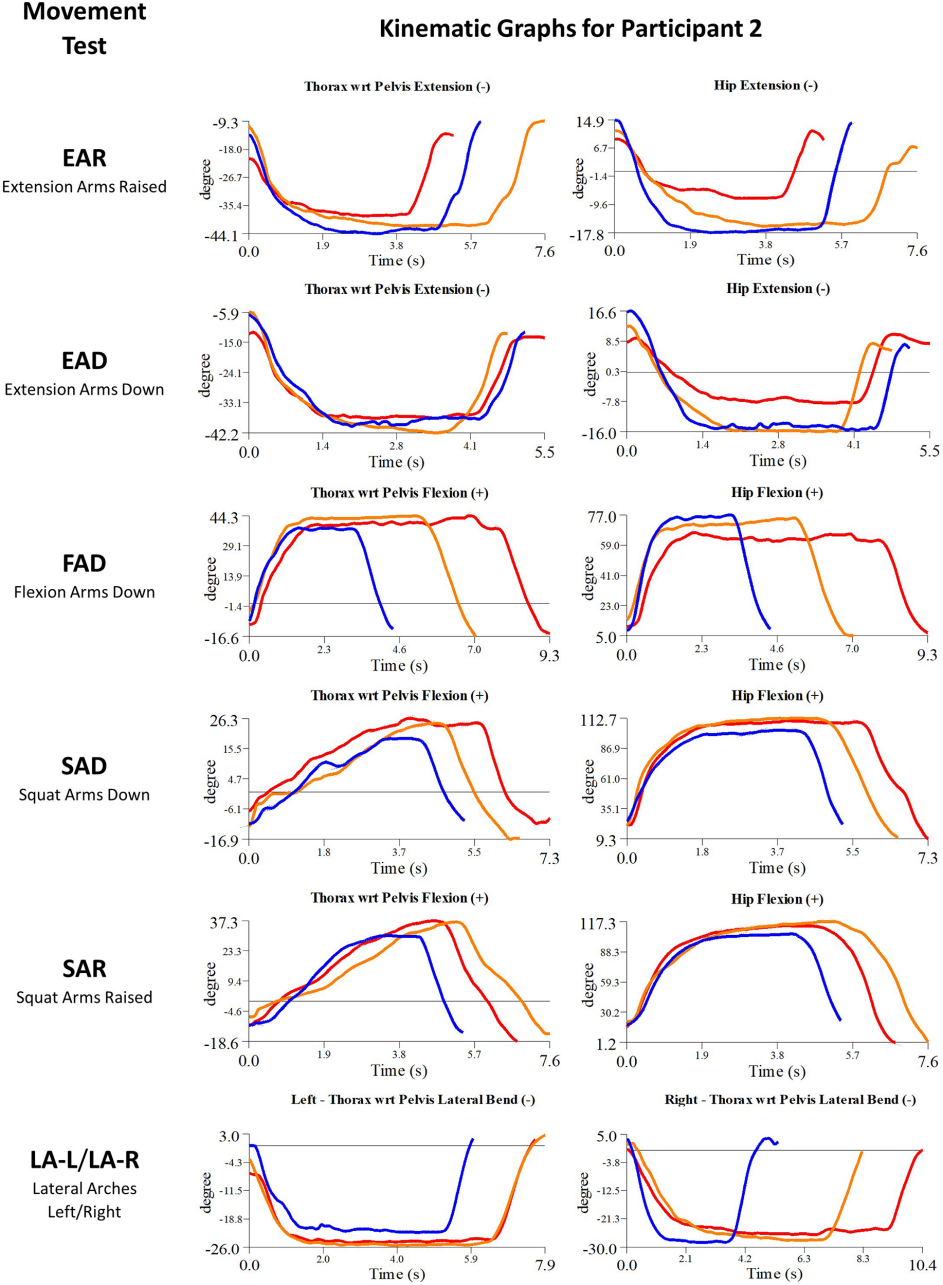
Participant 2 kinematics—single-plane joint angles plotted against time for the movement tests that were expected to change, or demonstrated unexpected changes; (i) baseline(red), (ii) post-first myoActivation session (orange), and (iii) post-final myoActivation session (blue).

**Figure 7:**
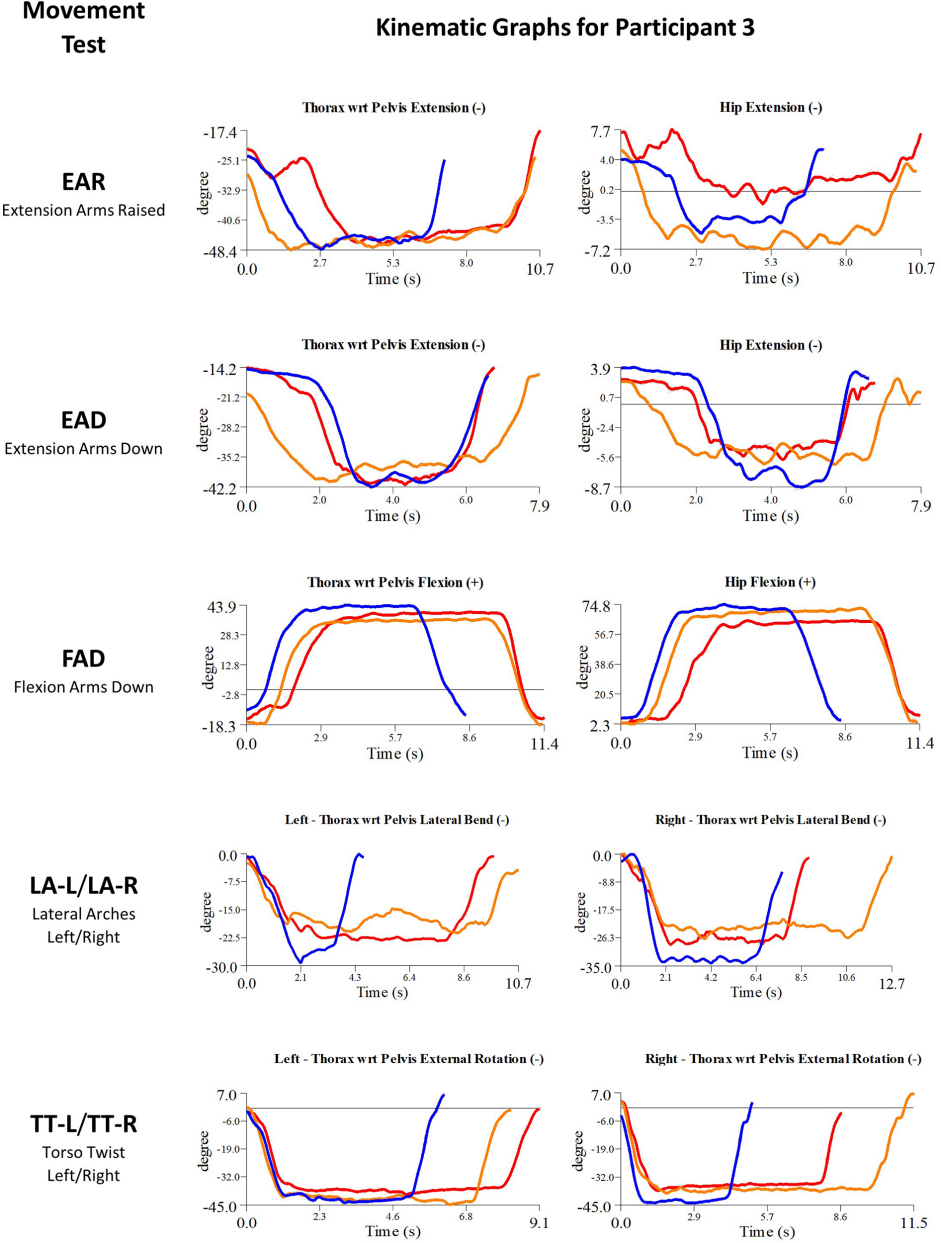
Participant 3 kinematics—single-plane joint angles plotted against time for the movement tests that were expected to change, or demonstrated unexpected changes; (i) baseline(red), (ii) post-first myoActivation session (orange), and (iii) post-final myoActivation session (blue).

Details of the initial treatment administered during the first myoActivation session are given in Table 5 and the relevant single-plane joint angle graphs for the core movement tests for all of study participants are shown in Figures 3–7.

**Table 5:** First myoActivation session; initial treatment details and the most likely expected changes (performed in the motion Lab).

ID	Reported pain site	Physician-observed initial worst BASE test		Muscles activated	Scars released	Areas where fascia in tension released	Movement tests most likely to change based on initial myofascial releases
		For pain	For ROM				
1	Right hip	SLS-R	SLS-R	Bilateral iliopsoas Right external oblique	Right shin Left knee	Right iliac crest	SAD SAR LA-L/LA-R
2	Left lower quadrant abdomen	LA-L LA-R	FAD	Bilateral iliopsoas Left external oblique Left rectus abdominus	Left knee chin	Left iliac crest Line of tension above umbilicus	EAD FAD SAD SAR LA-L/LA-R
3	Multisite and CLBP	EAR	FAD	Left iliopsoas Right paraspinal Left rectus abdominus Left external oblique Bilateral gluteus medius	None	Right iliac crest Line of tension above umbilicus	EAR EAD FAD LA-L/LA-R
4	Left hip and CLBP	TT-L TT-R	EAR	Left quadratus lumborum Bilateral rectus abdominus Left iliopsoas	Left thigh	Left flank skin crease Left iliac crest	EAD SAD TT-L/TT-R LA-L/LA-R
5	CLBP	EAR	EAR	Right paraspinal	None	Left iliac crest Coccyx	EAR FAD LA-L

CLBP, chronic low back pain; ROM, range of motion; EAR, extension arms raised; EAD, extension arms down; FAD, flexion arms down; SAD, squat arms down; SAR, squat arms raised; SLS, single leg squat; LA, lateral arch; TT, torso twist (see Figure 2).

CMA found evidence of improved maxROM and/or speedROM in 12/19 (63%), of the movement tests that the clinician had predicted would change after the initial myoActivation session (Table 6). Kinematic data corroborated the myoActivation physician observations of positive change in 9/11 (81%) movement tests in the initial TML session. Furthermore, CMA found objective evidence of improved maxROM and/or speedROM in 20/26 (77%) of the movement tests expected to change over the course of treatment (i.e., from baseline assessment to final TML assessment) (Table 7).

**Table 6:** Comparison of physician observed vs. objectively measured changes in movement tests for Initial myoActivation session in the Motion Lab.

Movement tests predicted to change		Objectively measured changes in kinematic data				Physician observation	Did kinematic data confirm physician observation of improvement
		Improved maxROM?	Increase in maxROM (bold indicates >5°)	Improved speedROM?	Increase in speedROM (bold indicates >5°/s)		
1	SAD	**Yes**	TWRP-F + 4; **HF +7**	SAD	**TWRP-F** **+** **8; HF +19**	**SAD improved**	**Yes**
	SAR	**Yes**	**TWRP-F** **+** **5**; HF +4	SAR	**TWRP-F** **+** **9; HF +29**	**SAR improved**	**Yes**
	LA-L, LA-R	**Yes**	**L** **+** **11; R** **+** **15**	LA-L, LA-R	**L** **+** **5; R** **+** **20**	**LA-L, LA-R improved**	**Yes**
2	EAD	**Yes**	TWRP-E + 4; **HE +6**	EAD	**TWRP-E** **+** **7; HE +9**	**EAD improved**	**Yes**
	FAD	**Yes**	TWRP-F 0; **HF +10**	FAD	TWRP-F + 2; **HF +9**	**FAD improved**	**Yes**
	SAD	No	TWRP-F −2; HF +2		TWRP-F + 2; HF +2	No change	** **
	SAR	No	TWRP-F −1; HF +4		TWRP-F −3; HF −14	No change	** **
	LA-L, LA-R	No	L 0; R + 2	LA-L, LA-R	L + 3; R + 1	**LA-L, LA-R improved**	No
3	EAR	**Yes**	TWRP-E + 2; **HE +5**		TWRP-E + 2; HE +2	No change	
	EAD	No	TWRP-E −1; HE −1		TWRP-E −6; HE −1	No change	** **
	FAD	**Yes**	TWRP-F −3; **HF +8**	FAD	TWRP-F + 4; **HF +12**	**FAD improved**	**Yes**
	LA-L, LA-R	No	L −2; R −2		L + 1; R + 1	No change	** **
4	EAD	No	TWRP-E 0; HE −1	EAD	**TWRP-E** **+** **7**; HE −2	**EAD improved**	**Yes**
	SAD	No	TWRP-F −6; HF +3	SAD	TWRP-F −3; HF +2	**SAD improved**	No
	LA-L, LA-R	**Yes**	**L** **+** **7; R** **+** **7**	** **	**L** **+** **2; R** **+** **12**	No change	
	TT-L, TT-R	No	L −10; R + 2		L −14; R + 1	No change	** **
5	EAR	**Yes**	**TWRP-E** **+** **13**; HE −3	EAR	**TWRP-E** **+** **7**; HE 0	**EAR improved**	**Yes**
	FAD	No	TWRP-F + 2; HF +1	** **	**TWRP-F** **+** **13**; HF +4	No change	
	LA-L	**Yes**	**L** **+** **6; R** **+** **9**	LA-L, LA-R	L + 3; **R** **+** **5**	**LA-L, LA-R improved**	**Yes**

maxROM, maximum range of motion; speedROM, maximum angular speed to maxROM. Improved maxROM and improved speedROM “yes/no” indicators were selected as “yes” if two joints were analyzed for a movement and either one or both demonstrated improvement. L, left; R, right; HE, hip extension; HF, hip flexion; TWRP-E, thorax with respect to pelvis—extension; TWRP-F, thorax with respect to pelvis—flexion; EAR, extension arms raised; EAD, extension arms down; FAD, flexion arms down; SAD, squat arms down; SAR, squat arms raised; SLS, single leg squat; LA, lateral arch; TT, torso twist (see Figure 2).

**Table 7:** Comparison of physician observed vs. objectively measured changes in movement tests between initial and final myoActivation sessions in the Motion Lab.

ID	Movement tests predicted to change	Objectively measured changes in kinematic data			
		Improved maxROM?	Increase in maxROM (bold indicates >5°)	Improved speedROM?	Increase in speedROM (bold indicates >5°/s)
1	EAD	**Yes**	**TWRP-E** **+** **17**; HE +3	**Yes**	**TWRP-E** **+** **9**; HE +1
	FAD	No	TWRP-F + 1; HF +4	**Yes**	**TWRP-F** **+** **29; HF +42**
	SAD	**Yes**	**TWRP-F** **+** **5; HF +7**	**Yes**	**TWRP-F** **+** **10; HF +40**
	SAR	**Yes**	TWRP-F + 2; **HF +11**	**Yes**	**TWRP-F** **+** **9; HF +48**
	LA-L, LA-R	**Yes**	**L** **+** **10; R** **+** **22**	**Yes**	**L** **+** **14; R** **+** **21**
2	EAD	**Yes**	TWRP-E + 3; **HE +7**	**Yes**	TWRP-E + 2; **HE +18**
	FAD	**Yes**	**TWRP F −6; HF +12**	**Yes**	TWRP F + 3; **HF +20**
	SAD	No	TWRP-F −7; HF −5	No	TWRP-F + 2; HF −14
	SAR	No	TWRP-F −7; HF −5	No	TWRP-F + 4; HF −9
	LA-L, LA-R	No	L −3; R + 3	**Yes**	L + 1; **R** **+** **14**
3	EAR	No	TWRP-E + 1; HE +3	No	TWRP-E −1; HE +1
	EAD	No	TWRP-E + 1; HE +2	No	TWRP-E + 3; HE +3
	FAD	**Yes**	TWRP-F + 4; **HF +10**	**Yes**	**TWRP-F** **+** **7; HF +15**
	LA-L, LA-R	**Yes**	**L** **+** **6; R** **+** **6**	**Yes**	**L** **+** **5; R** **+** **14**
	TT-L, TT-R	**Yes**	L + 4; **R** **+** **6**	No	L + 2; R + 3
4	EAR	**Yes**	TWRP-E −25; **HE +27**	**Yes**	**TWRP-E** **+** **5**; HE +2
	EAD	**Yes**	TWRP-E −27; **HE +25**	No	TWRP-E −1; HE 0
	FAD	**Yes**	**TWRP-F** **+** **19**; HF −15	**Yes**	TWRP-F + 1; **HF +16**
	SAD	No	TWRP-F + 4; HF −17	No	TWRP-F −5; HF −2
	LA-L, LA-R	**Yes**	L −2; **R** **+** **8**	**Yes**	**L** **+** **10**; R + 3
	TT-L, TT-R	No	L −2; R −16	No	L −4; R −12
5	EAR	**Yes**	**TWRP-E** **+** **7; HE +9**	**Yes**	**TWRP-E** **+** **5**; HE +4
	EAD	**Yes**	TWRP-E + 3; **HE +10**	**Yes**	**TWRP-E** **+** **14; HE +8**
	FAD	**Yes**	**TWRP-F** **+** **12**; HF −14	**Yes**	**TWRP-F** **+** **16**; HF −6
	SAD	**Yes**	**TWRP-F** **+** **14**; HF −2	**Yes**	**TWRP-F** **+** **10; HF +7**
	LA-L, LA-R	**Yes**	**L** **+** **9; L** **+** **8**	**Yes**	L + 4; **L** **+** **10**

maxROM, maximum range of motion; speedROM, maximum angular speed to maxROM; Improved maxROM and Improved speedROM “yes/no” indicators identified “yes” if two joints were analyzed and either one or both demonstrated improvement beyond threshold. L, left; R, right; HE, hip extension; HF, hip flexion; TWRP-E, thorax with respect to pelvis—extension; TWRP-F, thorax with respect to pelvis—flexion; EAR, extension arms raised; EAD, extension arms down; FAD, flexion arms down; SAD, squat arms down; SAR, squat arms raised; SLS, single leg squat; LA, lateral arch; TT, torso twist (see Figure 2).

#### 3.3.1. Kinematic data: baseline vs. after initial myoActivation session

##### 3.3.1.1. Participant 1

Participant 1 presented with pain in her right hip. The physician observed worst movement test for pain was SLS-R and worst movement tests for ROM were SLS-R, EAR, and LA (-L and -R). Based on these physician observations, myoActivation treatment was administered (see Table 5 for details). The movement tests the clinician predicted would change based on this treatment included SAD, SAR and LA (-L and -R).

Following treatment, the physician observed movement changes in 3/3 of these movement tests. Kinematic data provided objective evidence for improved maxROM in 3/3 of these tests, and improved speedROM in 3/3 of these tests. Kinematic data corroborated physician observations in 3/3 of these tests (Figure 5 and Table 6). FAD also showed a positive change in speed ROM that was not expected to change.

##### 3.3.1.2. Participant 2

Participant 2 presented with pain in the left lower quadrant of her abdomen. The physician observed worst movement test for pain were LA (-L and -R) and for ROM was FAD. Based on these observations, myoActivation treatment was administered (Table 5). The movement tests the clinician predicted would change based on this treatment included EAD, FAD, SAD, SAR, and LA (-L and -R).

Following treatment, the physician observed changes in 3/5 of these movement tests [EAD, FAD, and LA (-L and-R)]. Kinematic data provided objective evidence for improved maxROM in 2/5 of these tests (EAD and FAD), and improved speedROM in 2/5 of these tests (EAD and FAD). Kinematic data corroborated physician observations in 4/5 of these tests (EAD, FAD, SAD, SAR) (Figure 6 and Table 6). EAR showed a positive change in maxROM and speed ROM that was not expected to change.

##### 3.3.1.3. Participant 3

Participant 3 presented with multisite pain including chronic low back pain. The physician observed worst movement test for pain were EAR and LA (-L and -R), and for ROM were FAD and TT-L/R. Based on these observations, myoActivation treatment was administered (Table 5). The movement tests the clinician predicted would change based on this treatment included EAR, EAD, FAD, and LA (-L and -R).

Following treatment, the physician observed change in only 1/4 of these movement tests (FAD). Kinematic data provided objective evidence for improved maxROM in 2/4 of these tests (EAR, FAD), and improved speedROM in 1/4 of these tests (FAD). Kinematic data corroborated physician observations in 3/4 of these tests [EAD, FAD, LA (-L and -R)] (Figure 7 and Table 6).

##### 3.3.1.4. Participant 4

Participant 4 presented with left hip pain and chronic low back pain. The physician observed worst movement test for pain was TT, and for ROM was EAR. Based on these observations, myoActivation treatment was administered (Table 5). The movement tests the clinician predicted would change based on this treatment included EAD, SAD, and LA (-L and -R), TT (-L and -R).

**Figure 8:**
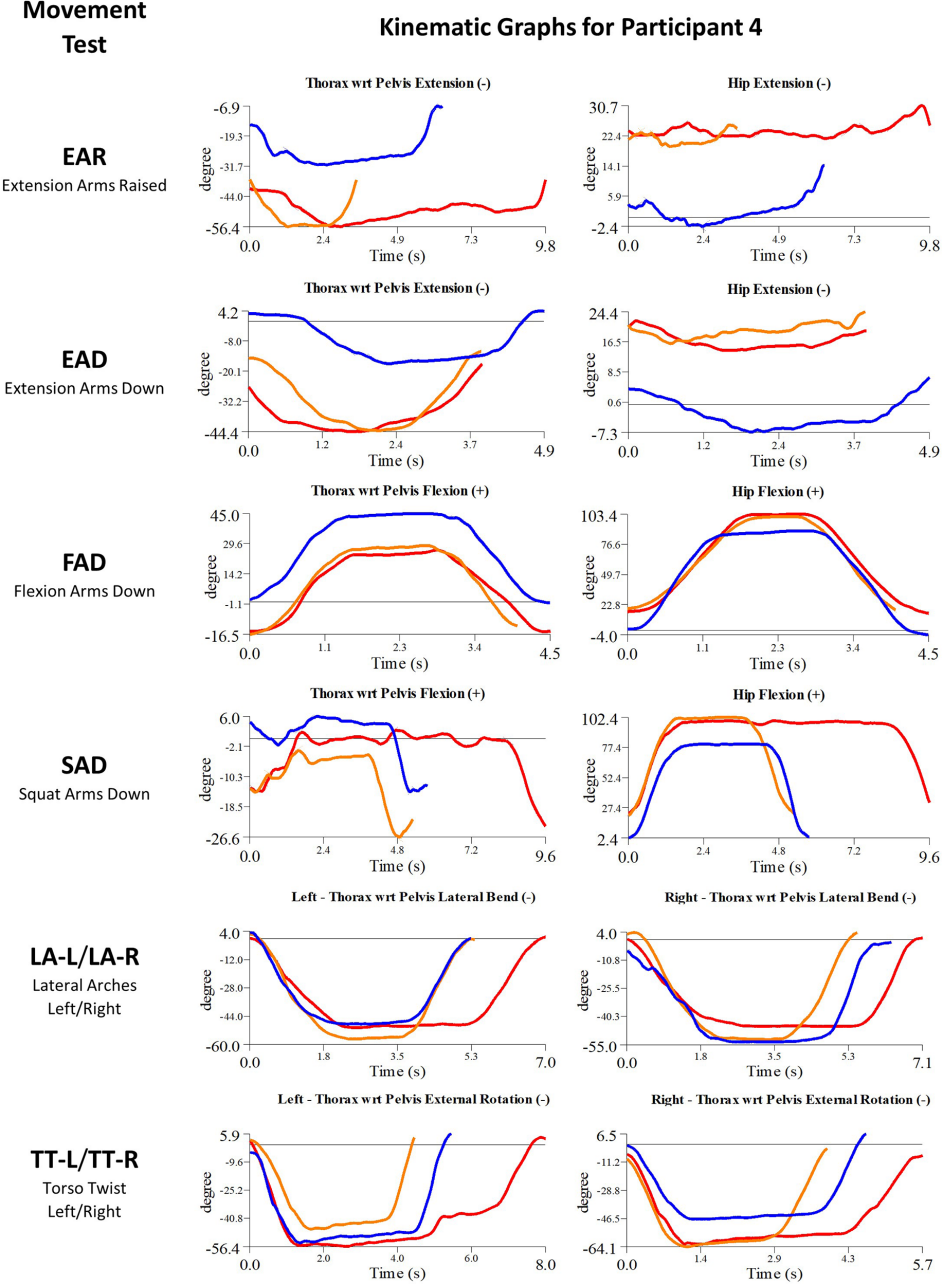
Participant 4 kinematics—single-plane joint angles plotted against time for the movement tests that were expected to change, or demonstrated unexpected changes; (i) baseline(red), (ii) post-first myoActivation session (orange), and (iii) post-final myoActivation session (blue).

Following treatment, the physician observed changes in 2/4 of these movement tests (EAD, SAD). Kinematic data provided objective evidence for improved maxROM in 1/4 of these tests [LA (-L and –R)], and improved speedROM in 2/4 of these tests (EAD, LA (-L and –R). Kinematic data corroborated physician observations in 2/4 of these tests [EAD, TT (-L and -R)] (Figure 8 and Table 6). EAR showed a positive change in speedROM and SAR improved maxROM, which were not expected to change.

##### 3.3.1.5. Participant 5

Participant 5 presented with chronic low back pain. The physician observed worst movement tests for pain was EAR, and for ROM were EAR and LA-L. Based on these observations, myoActivation treatment was administered (Table 5). The movement tests the clinician predicted would change based on this treatment included EAR, FAD and LA (-L and -R).

**Figure 9:**
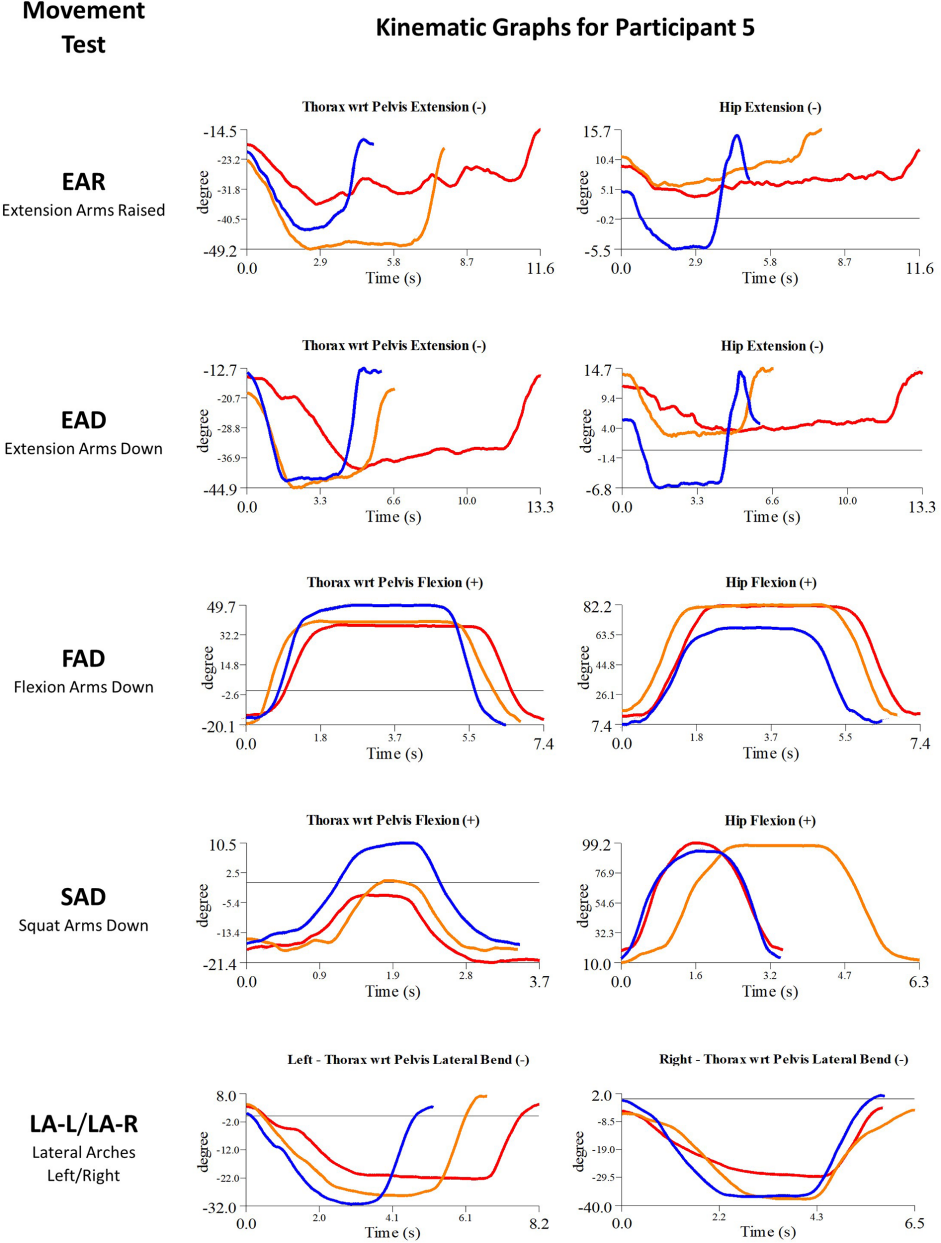
Participant 5—single-plane joint angles plotted against time for the movement tests that were expected to change, or demonstrated unexpected changes; (i) baseline(red), (ii) post-first myoActivation session (orange), and (iii) post-final myoActivation session (blue).

Following treatment, the physician observed change in 2/3 of these movement tests [EAR, LA (-L and -R)]. Kinematic data provided objective evidence for improved maxROM in 3/4 of these tests [EAR, FAD, LA (-L and -R)], and improved speedROM in 3/3 of these tests. Kinematic data corroborated physician observations in 2/3 of these tests [EAR, LA (-L and -R)] (Figure 9 and Table 6). EAD showed a positive change in maxROM and speed ROM that was not expected to change.

#### 3.3.2. Baseline vs. after myoActivation course

All participants received 2–5 myoActivation sessions as a course before the post-final myoActivation session timepoint (Table 3).

##### 3.3.2.1. Participant 1

Movement tests predicted to change based on the cumulative treatments over the course of the study included EAD, FAD, SAD, SAR and LA (-L and -R) (Table 7).

Kinematic data provided objective evidence for improved maxROM in 4/5 of these tests [EAD, SAD, SAR, LA (-L and –R)], and improved speedROM in 5/5 of these tests (Figure 5 and Table 7).

##### 3.3.2.2. Participant 2

Movement tests predicted to change based on the cumulative treatments over the course of the study included EAD, FAD, SAD, SAR and LA (-L and -R) (Table 7).

Kinematic data provided objective evidence for improved maxROM in 2/5 of these tests (EAD, FAD), and improved speedROM in (EAD, FAD, LA-R) in 3/5 of these tests (Figure 6 and Table 7).

##### 3.3.2.3. Participant 3

Movement tests predicted to change based on the cumulative treatments over the course of the study included EAR, EAD, FAD, LA (-L and -R) and TT (-L and -R) (Table 7).

Kinematic data provided objective evidence for improved maxROM in 3/5 of these tests [FAD, LA (-L and -R) and TT-R], and improved speedROM in 2/5 of these tests [FAD, LA (-L and -R)] (Figure 7 and Table 7).

##### 3.3.2.4. Participant 4

Movement tests predicted to change based on the cumulative treatments over the course of the study included EAR, EAD, FAD, SAD, LA (-L and -R) and TT (-L and -R) (Table 7).

Kinematic data provided objective evidence for improved maxROM in 4/6 of these tests (EAR, EAD, FAD, LA-R) and improved speedROM in 3/6 of these tests (EAR, FAD, LA-L) (Figure 8 and Table 7).

##### 3.3.2.5. Participant 5

Movement tests predicted to change based on the cumulative treatments over the course of the study included EAR, EAD, FAD, SAD and LA (-L and -R) (Table 7).

Kinematic data provided objective evidence for improved maxROM and improved speedROM in 5/5 of these tests (Figure 9 and Table 7). A video of the single-plane skeletal model demonstrates the changes for the EAD core movement test for participant 5, showing (i) baseline, (ii) post-first myoActivation session, and (iii) post-final myoActivation session (Supplementary Material Video S1).

### 3.4. Changes in self-reported painful movements

Participants reported a median (range) of 6 (1–7) painful movements in their first session pre-intervention and 1 (0–6) painful movements at the end of their myoActivation course. Four participants had a reduced number of painful movement tests, but participant #3 did not.

## 4. Discussion

This exploratory feasibility study has provided preliminary evidence of beneficial changes in maximum range of motion (maxROM) and/or movement speed to reach maxROM (speedROM) in adolescent patients who underwent myoActivation for chronic pain related to myofascial dysfunction. Five participants received myoActivation assessment and therapy, with at least the first baseline movement tests and last follow-up movement tests observed with motion capture technology to support the detailed quantitative analyses presented.

The kinematic data demonstrated evident movement changes to varying degrees, as indicated by increased maxROM and speedROM, for all participants over both time intervals. These changes were largely consistent with physician expectations based on the treatment administered. The CMA generated objective metrics indicating positive movement changes in 12/19 (63%) of the movement tests that the physician expected to change in the initial myoActivation session. The maxROM and/or speedROM were improved in 20/26 (77%) movement tests that were predicted to change from baseline to final TML.

This provides evidence that myoActivation can be effective in promoting improved movement. Our data suggests the improvements may be predictable based on the interventions provided, and that the improvements detected by CMA are fairly consistent with clinical observation. These findings are promising and suggest that a larger study of this assessment and treatment system is worthwhile. CMA may have the potential to guide myoActivation treatment by objectively automating some of the assessment process and aiding in the diagnosis of myofascial dysfunction. Importantly, CMA may have a role in supporting the ongoing teaching and learning of the myoActivation technique.

### 4.1. Clinical interpretation

myoActivation is unique in following a set of structured movement tests to determine the most important tissues requiring treatment. The physician is looking for a movement test which stands out over all others with respect to pain, movement restriction or both. When tissues in this target area are treated, the movement test that was most restricted or painful is most likely to change. This was demonstrated well in participants 1 and 5 after the intitial myoActivation session. After each individual myofascial area is treated, movement tests are repeated to demonstrate this change and direct the clinician to the next most important target area. Several cycles occur during each myoActivation session. The purpose of these catenated cycles is to help unravel multiple sources that contribute to the full myofascial pain presentation. Immediate treatment responses occur, which include reduction in pain, observed increased flexibility, and improved fluidity of movement; these responses were observed in 4 of our 5 participants.

Humans exhibit biotensegrity, whereby the whole body functions as a three-dimensional visco-elastic vehicle no matter what position it adopts. In this system the skeletal bones are non-contact compression struts embedded in a networked and tensioned myofascial matrix. Biotensegrity dictates that each individual part of the organism combines with the mechanical system to create one integrated functional movement unit. Each component of a biotensegral structure contributes to the stability of the whole system. Tensegral structures, like a suspension bridge, are strong, light-weight, flexible, resilient, move with minimal effort, and return to the same position. Biotensegrity requires a reconceptualization of anatomy and an understanding that myofascial stiffness generated by muscles in sustained contraction, scars and fascial density generate anomalous tension affecting the whole fascial continuum leading to progressive immobility and pain (7, 8). This is not just a local phenomenon because ROM at one peripheral articulation affects the whole body-wide kinematic system (32). This is demonstrated by beneficial changes seen in movements that were not expected to change in 4 of our 5 participants after the initial myoActivation session.

### 4.2. Use of motion capture technology to generate evidence for chronic pain therapies

In the current study, the kinematic metrics that reflected changes in ROM in adolescent patients undergoing myoActivation treatment for CMP included maximum ROM (maxROM) and maximum angular speed to achieve maximum ROM (speedROM). The utilization of motion capture technology to quantify the joint angles and movement speed has yielded data that can be used to objectively gauge the effect of the myoActivation therapeutic interventions on participant performance of the structured myoActivation movement tests. The data captured indicate that the change in maxROM and speedROM that each participant can achieve in each movement test following myoActivation, can vary substantially. Factors that may influence these changes include initial flexibility of the participants and the type and number of myoActivation interventions deemed necessary for each individual participant. Further investigation is needed to determine how kinematic metrics correlate with specific myoActivation interventions.

The speedROM metric in this study provided a measure of the movement speed that participants self-selected to perform the myoActivation movement tests. Participants demonstrated an increase from baseline in speedROM in various movement tests over both the first-session interval and the full-study interval, which suggests greater comfort of the participant during the movement tests. While speedROM is a valid approximation of movement speed, there is scope to further investigate the participant's speed to achieve maximum ROM in each movement, particularly when the movements are not smooth. Less smooth movements may be an indicator of lack of stability, discomfort, or other internal physiological factors, all of which may be affected by the myoActivation treatments provided.

Completion of myoActivation assessment and treatment sessions in conjunction with collection of CMA data was feasible. Patients underwent multiple rounds of intervention in TML, which was well tolerated with good interventional outcomes except for participant 3. myoActivation could be completed with the presence of reflective markers placed on key bony landmarks. Ease of data collection could be further optimized with utilization of markerless motion capture technology. Further work is needed to optimize timing of CMA data collection (after all treatments vs. with each treatment). It is important to observe that myoActivation is a process whereby myofascial tissues are treated sequentially, hence the need for 2–4 sessions in a course of treatment for complete resolution.

Based on the current study, further detailed exploration of the effects of myoActivation on maximum ROM and maximum angular speed to reach maximum ROM, are recommended. For example, collection and comparison of motion capture data between individual myoActivation therapeutic interventions would facilitate a more granular appreciation of the direct effects of each therapeutic intervention on any ensuing change in kinematic metrics. CMA may also help determine which patients may not have a major component of myofascial dysfunction contributing to their pain, where movement tests do not improve as expected with myoActivation.

There are three other potential avenues we can investigate to determine how CMA may be applied clinically in this context. Firstly, CMA may be able to support and refine the assessment process. Observing movement tests is complex and determining the appropriate course of treatment based on those observations can be challenging; if the kinematic data can be processed in near real-time, then clinicians could obtain objective point-of-care information to corroborate and enhance their own observations. CMA may also capture nuances in the movement tests that the clinician fails to notice; and it is even possible, if not ideal, that movement tests could be performed in the absence of direct clinician observation. Secondly, CMA can support the trainee or less experienced myoActivation practitioner; the newly-acquired techniques of myoActivation may be more confidently applied if supported by objective point-of-care data that are available in real-time. Thirdly, there is scope for integrating these complex data into a machine learning assisted system that could identify patterns in the complex movement data that are not recognized by the human clinician. This could confirm outcomes of specific treatment targets, enable more finely tuned treatment decisions, reduce needling sessions, and suggest alternative target areas for treatment.

### 4.3. Limitations

We acknowledge some limitations with this present study. As a small feasability study, the findings cannot be generalized to support firm recommendations for the use of myoActivation in this population; rather, the current study has established the feasibility of using CMA to objectively measure observed clinical changes. Additionally, all participants were female; they are more common than males within the CPS at BCCH and therefore more frequently recommended for myoActivation assessment and treatment.

Motion capture was not used for every myoActivation session; this was a practical limitation based on scheduling with families, clinical staff, and the availability of TML. We were aware of this at the outset and resolved that a minimum requirement of first baseline movement tests and last follow-up movement tests with motion capture would be acceptable to establish the feasibility of our approach.

Participants were not asked to do each movement test multiple times during each motion CMA session, which would have accounted for variability in the participants' performance of the movement tests and established a baseline average, because the movements often induce pain. However, the initial myoActivation movement test was the third time participants were performing these tests. The movement tests in this study were administered according to a script; however, participants were not instructed to perform the movement tests at any particular speed (e.g., “as fast as you can” or “for a certain duration of time”), but rather were undirected and were permitted to move at their self-selected speed. Multiple participants demonstrated an ability to reach greater speeds towards their maximum ROM after interventions than before, as evidenced by the speedROM results. However, providing guidance about performing the movement tests at certain speeds may have yielded different speedROM data. In future work, participants will be given more detailed instructions about movement speed and/or about when to start the movement test.

Kinematic data collected via motion capture methods are based on the location of the reflective markers placed on anatomical landmarks. Since the participants had reflective markers placed on their body on two or more separate visits for the time-points presented in the current study, there is potential for discrepancy of the location of the reflective markers between time-points. In the current study, the “evident change” thresholds for the kinematic data were set at 5° for maxROM and 5°/s for speedROM, which is slightly greater than the typical uncertainty error of 3° for TWRP and hip angle measurements in TML during gait analyses, suggesting that the current study employed an appropriately conservative estimate of uncertainty. However, for participant 4, based on the TWRP and hip joint data observed over the second time interval in which the TWRP and HE exhibited opposite and similar magnitude changes in maxROM in the sagittal plane (Table 7), there is reason to think that the pelvis markers were applied inconsistently between sessions in TML. In order to quantify marker placement-related errors for the movements analyzed in the current study, a repeatability analysis of the marker placement and associated uncertainty error of measured kinematic data (i.e., joint angles and joint angular speed) is recommended for future studies.

myoActivation interventions performed at the first session are usually restricted to a small number of needle insertions to minimize discomfort and maintain a therapeutic trust and alliance between physician and patient. First session interventions may also be limited if there is an emotional or potential vasovagal response (light headedness, pallor, or excessive sweating) to the needling technique. It is possible that limiting numbers of needle insertions for the first intervention could result in lack of effective release of the affected tissue. Major scars will usually only be released if the scar has been pre-treated with a topical anesthetic agent. If this topical agent has been omitted then scar release will be deferred to the next session so that the topical anesthetic can be applied. Release of fascial tension at the coccyx is often deferred until a second session to establish trust before exposing a private area for treatment. All these factors may restrict the full potential of the first myoActivation session.

### 4.4. Conclusion

This study utilized motion capture technology to provide preliminary, but compelling, evidence of beneficial changes in range of motion in adolescent patients undergoing myoActivation for chronic pain related to myofascial dysfunction. This novel approach facilitated objective measures of outcomes that are typically evaluated subjectively by a clinical observer and may have broad applicability in evaluating chronic pain therapies. Further research is required to firmly establish the objective benefit of myoActivation therapy, in the form of a larger prospective clinical trial using the same observation techniques and outcome measures established in this feasibility study.

## Data Availability

The datasets presented in this article are not readily available because ethical approval for data sharing was not requested and not included in patient consent. Requests to access the datasets should be directed to glauder@cw.bc.ca.

## Supplementary material

Supplementary Material Video S1 Single-plane video of skeletal model of one core movement test (EAD) for participant 5, showing (i) baseline, (ii) post-first myoActivation session, and (iii) post-final myoActivation session.
