# Supplementary material for: Assessing changes in range of motion in adolescent patients undergoing myoActivation® for chronic pain related to myofascial dysfunction: a feasibility study
Source: Front Pain Res (Lausanne). 2023 Oct 25;4:1225088. doi: 10.3389/fpain.2023.1225088 (PMC10634437; doi:10.3389/fpain.2023.1225088)
Supplement: Supplementary file 2 [file Datasheet1.pdf]

myoActivation Scripts for Movement Tests 2022

Purple = instructions to the patient

Black = notes/instructions for observers

Red = tasks and cues for TML personnel

With any movements that appear severely restricted, add "Can you go further?"

Once the patient is ready to have reflective stickers applied, the TML physiotherapist will obtain height and weight measurements and report them to Tim/Mona.

When the patient comes into the lab after having their reflective stickers applied, Tim/Mona will:

- Guide the patient to a specific location in the middle of the lab
- Ask the patient to stand, with arms slightly elevated, and hold the pose for ~5sec
- Ask the patient to perform a 'light squat' and return to standing
- The TML physio will then remove the 'extra' reflective stickers

***These tasks will only be performed once, per patient visit to the lab*

BASE Test	Code	Script for Movement Test
Initial Pain Site		Stand facing me. Put a fingertip on where you feel the most pain, if you do have any pain?
**bal**ance/weight on feet	BAL	Tim/Mona will guide the patient to stand in front of the pressure mat, in anticipation of the BAL test. When the system is ready, Tim/Mona will instruct the patient to step, with both feet, onto the pressure mat.<br><br>Which foot has more weight? right versus left, or is it 50/50?<br>Is there more weight on the backs or fronts of your feet, or is it central?<br>Is there more weight on the insides or outsides of your feet, or is it central?<br><br>Tim/Mona will then instruct the patient to stand on the forceplates, with one foot on each plate. Then the BAL test will be repeated<br><br>Which foot has more weight? right versus left, or is it 50/50?<br>Is there more weight on the backs or fronts of your feet, or is it central?<br>Is there more weight on the insides or outsides of your feet, or is it central?
**e**xtension **a**rms **r**aised	EAR	With your arms overhead, [pause until thearms are raised] push your hands back toarch back, bending from your hip joints as if you are going to look at the wall behind you to where you can **comfortably**.<br>What are you feeling?<br>The head should extend fully into cervicalextension<br>knees should be relatively straight (not flexed appreciably) so that pelvic rotationcontributes to spinal extension.
**e**xtension **a**rms **d**own	EAD	With your arms by your side, [pauseuntil the arms are in position] arch back, bending from your hip joints to where you can **comfortably**.<br>What are you feeling?

		Correct patient accommodations and ‘cheating’ if observed, such as:<br>• the head should extend fully intocervical extension<br>• knees should be relatively straight (not flexed appreciably) so that pelvic rotation contributes to spinalextension
**f**lexion **a**rms **d**own	FAD	With **straight** knees, flex forward, to touch your knees, shins, or floor – wherever you can go **comfortably**.<br>Emphasize the word ‘comfortably’. While patient is flexing forward, ask:<br>• Any discomfort in your back or neck?<br>Pain or tension in the legs is a normal restriction, whereas pain at any point above the ilium is not.
**s**quat **a**rms **d**own	SAD	With your arms by your side, [clinician to demonstratesquat with your arms by your side – try to demonstrate that hands should be slightly away from the body] squat to where you can **comfortably**.<br>What are you feeling?<br>If the pain is perceived to be in the upper leg, then the quadriceps will be the pain source.<br>If pain in the lower leg, then the gastrocnemius and/or soleus is the source<br>If a patient has a very restricted squat, their technique in performing the squat is likely a contributor to the lack of depth in movement.<br>When the clinician observes poor depth to the squat, consider having the patient repeat the squat with the following instruction:<br>• Push your buttocks backwards [helpful for the clinician to motion with their index finger in the direction for the buttocks to move] as you bend your knees<br>A deeper squat will invariably result due to increased pelvic rotation from driving the buttocks backwards.
**s**quat **a**rms **r**aised	SAR	With your arms overhead, squat to where you can **comfortably**.<br>What are you feeling?<br>If the pain is perceived to be in the upper leg, then the hamstrings will be the pain source. Ifin the lower leg, then the source is the tibial fascia or anterior compartment muscles.<br>If a patient has a very restricted squat, their technique in performing the squat is likely a contributor to the lack of depth in movement.<br>When the clinician observes poor depth to thesquat, consider having the patient repeat the squat with the following instruction:<br>• Push your buttocks backwards [helpful for the clinician to motion with their index finger in the direction for the buttocks to move] as you bend your knees<br>A deeper squat will invariably result due to increased pelvic rotation from driving the buttocks backwards.
**t**orso **t**wist	RTT<br>LTT	Cross your arms on your shoulders. I am going to brace your hips. The clinician should place their hands on thepatient’s lateral hips to prevent pelvic rotation. It is easier to brace if sitting on a stool or kneeling on one knee.<br>Rotate to one side. Any pain or discomfort? Rotate to the other side.Any pain or discomfort?<br>An alternative assessment posture is tohave the patient sit and

		perform rotation of the shoulders. This assessment posture does not provide the added information to the clinician that comes from feeling the torque on the bracing hands.
**l**ateral **a**rches	RLA<br>LLA	With one arm overhead, arch sideways like a ballerina.<br>Where do you feel pain or tension?
single **l**eg **b**alance	RLB<br>LLB	Stand on one leg and then theother. Which leg is more unsteady?<br>For patients who are not comfortable in independent single leg balance, the patient should hold the clinician's hands in preference to bracing on the wall or on a counter. The clinician gains valuable information on how the patient pulls or presses on the clinician's hand.
**pelvic** circumduction	PCD	Imagine you are doing a "hoola hoop" manoeuvre. Trying doing that with your pelvis but keep your chest and legs stable.<br>Do you feel restricted doing that movement?<br>If yes, where?<br>The clinician should demonstrate this movement. The clinician gains valuable information on where in the circular motion that patient is restricted or where the patient feels restricted.
single **leg** **squat**	RLS<br>LLS	With your arms by your side, squat on one leg to where youcan comfortably. I can hold your hand to steady you if you need.<br>The clinician gains valuable information on the degree of unsteadiness of one side compared to the other.
